# PReS-FINAL-2104: Disease course and predictors of outcome in systemic onset juvenile idiopathic arthritis

**DOI:** 10.1186/1546-0096-11-S2-P116

**Published:** 2013-12-05

**Authors:** S Soponkanaporn, S Vilaiyuk

**Affiliations:** 1 Pediatrics, Faculty of Medicine Ramathibodi Hospital, Bangkok, Thailand

## Introduction

Systemic juvenile idiopathic arthritis (SJIA) is the most common subtype of JIA in Thailand, which represents approximately 33 percents of all JIA patients. The disease course of SJIA and outcome of treatment were varied, depending on multi-factors. In the previous studies, persistent systemic features, younger age at onset, and arthritis of hip are predictors of poor functional outcome in SJIA. Since the development of biologic agents in this era improves the quality of life and outcome in SJIA patients, the disease course and predictors of outcome in SJIA have changed in the past 10 years.

## Objectives

To evaluate disease status, functional outcome, structural damage, and prognostic factors of SJIA outcome.

## Methods

Patients who were diagnosed with SJIA according to International League of Associations for Rheumatology (ILAR) criteria in Ramathibodi hospital between April 1997 and April 2013 were enrolled in this study and data records were reviewed. Disease status was evaluated by using European League Against Rheumatism (EULAR). Functional outcome was assessed by American College of Rheumatology core set criteria, including Child Health Assessment Questionnaire (CHAQ), physician global assessment, parent/patient global assessment, number of active joints, number of limited joints, and erythrocyte sedimentation rate (ESR), while radiographs were interpreted by radiologist using Dale radiographic classification system for structural damage evaluation. Prognostic factors of moderate to severe disability (defined using a CHAQ score ≥0.75) and moderate to severe radiographic damage (defined using Dale radiographic classification grading ≥3) were analyzed by multivariable logistic regression models.

## Results

Fifty-two patients (24 boys and 28 girls) were enrolled. The mean age of onset was 5.4 years old (6 months - 14.1 years). The mean follow up period was 43.1 months (6.3-169.6 months). The most common presentations were fever (100%), arthritis (100%) and salmon rash (75%). The patterns of arthritis were 55.8% polyarthritis and 44.2% oligoarthritis. The most commonly involved joints were the wrist (69.2%) and knee (67.3%). Treatment included systemic steroid (88.5%), disease-modifying antirheumatic drugs (86.5%), and biologic agents (42.3%). Among patients taking biologic agents, five received etanercept, one received infliximab, and fifteen received tocilizumab. Nine patients achieved complete remission, one patient with clinical inactive, thirty-three patients had stable disease, and six patients still had active disease. Six patients (12%) developed macrophage activation syndrome (MAS) during the disease course. Two patients died from MAS and severe infection, one patient died from severe infection. Seven patients (13.5%) had moderate to severe disability. The predictor of a CHAQ score ≥0.75 was initial presentation of polyarthritis (p 0.012). Eleven patients (32.4%) had moderate to severe radiographic damage and neutrophil counts at presentation ≥ 12000 cells/mm^3 ^was the only significant predictor for structural damage (p 0.028).

## Conclusion

Polyarthritis at initial presentation may predict the development of a poor functional outcome. The prognostic factor of radiographic damage was neutrophilia. Infection is the major cause of death in SJIA.

## Disclosure of interest

None declared.

